# Global Citizenship in Clinical Training: A Qualitative Study Evaluating a Co‐Creation Approach to Community Oral Health Interventions

**DOI:** 10.1111/eje.70076

**Published:** 2025-11-14

**Authors:** Mahdi Hussaini, Andrea Rodriguez, Clement Seeballuck

**Affiliations:** ^1^ University of Dundee Dundee UK

**Keywords:** co‐creation, dental education, oral health inequalities, vulnerable groups

## Abstract

**Introduction:**

Oral health plays a crucial role in overall well‐being, impacting general health, psychological health and quality of life. Vulnerable groups often face higher rates of oral health problems, exacerbating inequalities. This study evaluates the effectiveness of student‐delivered oral health interventions with these groups using a co‐creation approach.

**Materials and Methods:**

A qualitative study was conducted with 19 participants comprising dental students from the Dundee Dental Connect (DDC) project, their supervisors and third sector practitioners/managers working with vulnerable populations in Scotland. Online focus groups were conducted followed by thematic analysis with the assistance of NVivo software.

**Results:**

Seven major themes emerged from the analysis: enhanced student competence and confidence, support structures and training, evolving attitudes towards marginalised groups, community interaction, student engagement, challenges in community engagement and project sustainability. Students reported improved communication skills, confidence and clinical application, with co‐creation fostering empathy and emphasizing preventive dentistry.

**Discussion:**

The findings highlight the benefits of co‐creation in student‐delivered oral health interventions and dental education, enhancing student self‐efficacy and professional competencies when engaging with underserved populations. Challenges including language barriers were addressed through adaptability and mentorship. The study also underscores the importance of sustainable resource management and broader outreach to tackle health inequalities.

**Conclusion:**

The DDC project demonstrates the positive impact of co‐creation in dental education, fostering competencies and empathy among students, and preparing them to address oral health disparities in different settings. This approach strengthens community partnerships and equips future professionals with the skills to promote sustainable, equity‐driven oral healthcare.

## 1 Introduction

These are exciting times in undergraduate clinical training. Modern pedagogy continuously evolves, challenging and improving teaching methods [1]. Lessons learned from the pandemic and subsequent recovery periods have provided an opportunity for reflection for educators, institutions and professionals [2]. Inequality is still ubiquitous, with energy and cost of living crises highlighting this. In the UK, the shift from the General Dental Council from the existing Preparing for Practice [3] intended learning outcomes to the New Safe Practitioner framework [4] reflects the importance of social awareness and global citizenship when training future dentists.

Oral health is crucial for overall well‐being, extending far beyond merely having healthy teeth and gums. Poor oral health can significantly impact general health, quality of life and social interactions, affecting individuals across all aspects of their daily lives [5]. Numerous studies have consistently shown a strong and intricate link between oral diseases and various systemic conditions [6, 7].

This profound connection underscores the critical importance of maintaining good oral health for overall health and longevity, emphasising the need for a holistic approach to healthcare that integrates oral health into general medical care [8]. A complex interplay of factors, including but not limited to low income, limited access to dental care facilities, lack of comprehensive oral health education, and cultural barriers, contribute to significantly higher rates of oral diseases in these underserved populations [9, 10].

Traditional approaches to addressing these deeply rooted disparities, such as focusing solely on individual behaviour change or increasing the number of dental professionals, often fall short as they fail to adequately account for the myriad systemic barriers including persistent low socio‐economic backgrounds, chronic lack of access to affordable dental care, prohibitive cost issues and diverse cultural challenges that require sensitive and tailored interventions [11, 12].

In Scotland, these challenges are particularly evident and pressing. The Scottish Index of Multiple Deprivation (SIMD) data starkly highlights significant regional inequalities in both general and oral health outcomes. People living in the poorest areas of Scotland face the grim reality of dying on average 7 years earlier than their counterparts in the most affluent areas, while spending approximately 17 more years living with disabilities [13]. This stark combination of reduced life expectancy and prolonged periods of ill health underscores the critical need for comprehensive interventions.

The disparity in oral health is particularly evident among children. In Scotland, the NHS Tayside data reveals that 56% of children the most deprived areas are caries‐free compared to 86% in the least deprived areas, demonstrating a substantial 30% of inequality gap [14].

The disparity in oral health is not only evident among children but also persists into adulthood, reinforcing long‐standing inequalities. In Scotland, NHS data reveals a clear socioeconomic divide in dental care access and participation. As of September 30, 2022, only 42.7% of adults from the most deprived areas had attended a dental appointment within the past 2 years, compared to 53.5% in the least deprived areas, marking a stark disparity of 10.8 percentage points. This gap has widened significantly over time, increasing from just three percentage points in 2008 to its current level, underscoring the persistent barriers faced by adults in lower socioeconomic groups when accessing essential oral healthcare. These findings highlight the urgent need for targeted interventions to bridge the divide and promote equitable access to dental services across all communities [15].

Co‐design and co‐production methodologies in higher education, where students and educators work collaboratively to create curricula, develop teaching methods and define intended learning outcomes, are promising strategies that can revolutionise both education and community health interventions. These participatory approaches not only improve the educational experience by making it more relevant and engaging but also deeply engage students in the learning process, harbouring a sense of ownership and promoting democratic values in learning environments [16, 17].

The Dundee Dental Connect, student‐staff co‐creation initiative was established in 2023 to raise awareness of global citizenship and social purpose within students through initiatives that mitigate health inequalities of different groups in Dundee/Scotland. Prior to the pandemic, the Dundee School of Dentistry engaged with communities via an initiative called Toothy tigers‐ a predominantly student‐led endeavour [18].

Dundee Dental Connect was borne from an awareness of the changing environment and the widening inequalities ubiquitous in the post‐pandemic world and modern educational theory. Sustained oral health promotion initiatives have been developed in partnership with local organisations (foodbanks, homelessness services and educational projects) addressing the health and social care needs of homeless people, refugees and immigrants [19, 20, 21]. Co‐creation, the staff‐student partnership, is a method of adult learning that has grown in popularity in recent years and offers a number of advantages over traditional didactic teaching [22]. In this context, the Dundee Dental Connect (DDC) project exemplifies a co‐creation model, where students and staff collaborate in designing and delivering oral health interventions.

This paper evaluates the effectiveness of student‐delivered oral health interventions in addressing inequalities in the context of DDC, by using an innovative co‐creation approach, within cross sector collaboration to improve community health outcomes.

## 2 Aims and Objectives of the Study

1. To explore the impact of student‐delivered oral health interventions on the self‐efficacy of the student participants.
2. To explore the potential impact of student‐delivered oral health interventions on the attitudes of the student participants towards community engagement and oral health promotion.
3. Examining the influence of the co‐creation approach on the student perceived effectiveness of the interventions.
4. To provide evidence‐based insights and recommendations for the improvement and future development of student‐delivered oral health interventions.

## 3 Methodology

The study conducted a qualitative methodology through focus groups with the three groups of participants (students, supervisors and third‐sector managers) and employed thematic analysis with assistance of NVivo Software [23] to identify key patterns [24]. Focus groups were chosen as the primary data collection method as they can generate rich, nuanced data through participant interaction [25, 26]. This approach allows for dynamic discussions and the emergence of diverse viewpoints [27].

### 3.1 Participant Recruitment

**FIGURE 1:**
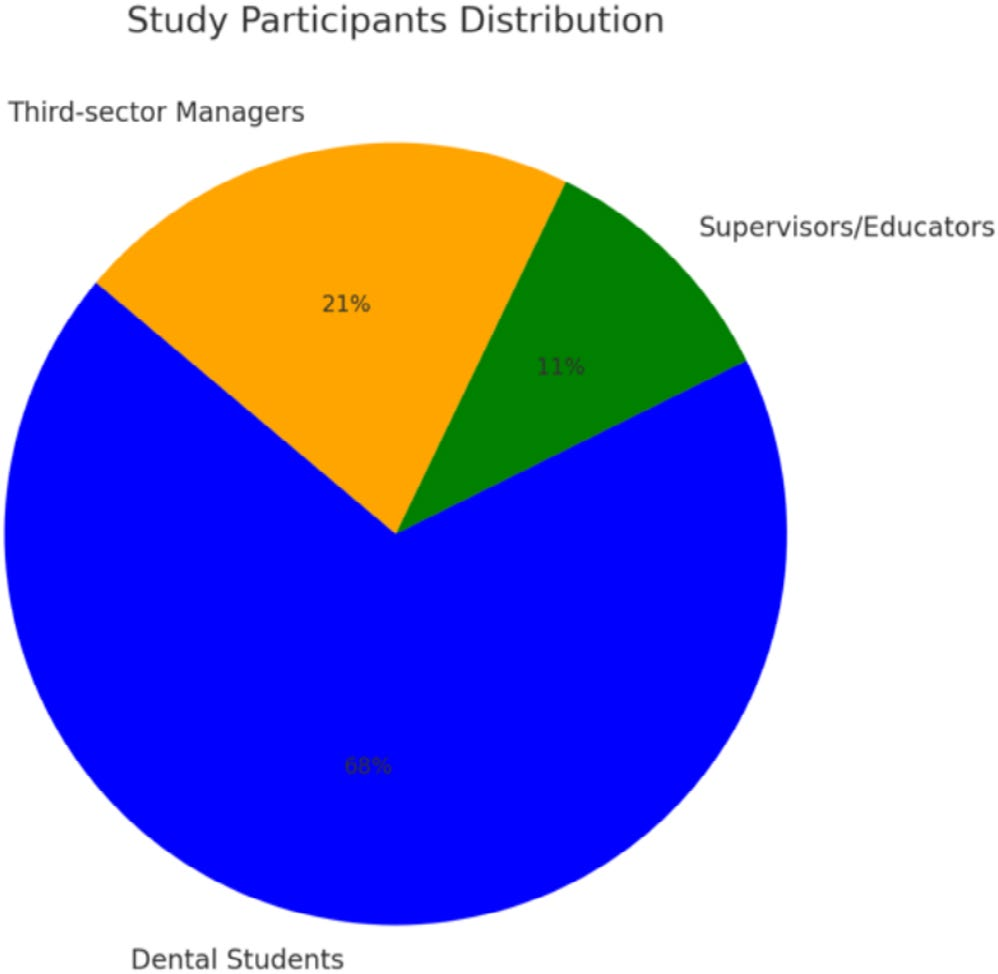
Study participants distribution.

The recruitment process was led by the principal investigator (MH). The study recruited 19 participants from three groups within the Dundee Dental Connect (DDC) project (Figure 1):

1. 13 Dental students.
2. 2 Supervisors/educators involved in the project.
3. 4 third‐sector managers involved in supporting marginalised groups in Dundee.

The dental students were selected through a randomised process, ensuring an unbiased representation of those actively participating in the DDC project. Supervisors and third‐sector managers were selected using purposive sampling, with invitations extended based on their involvement in the DDC project and their expertise in student training or community health services. Five supervisors/educators were initially invited to participate and two took part in the study. Six third‐sector managers from organisations involved in the oral health interventions delivered by DDC were approached to participate, and four agreed to participate. Two of them were unable to attend the focus groups on the planned date and provided their responses via email. This content was subsequently integrated into the thematic analysis.

All participants received comprehensive information sheets and informed consent forms, detailing the study's objectives, data protection measures and their rights. The voluntary nature of participation was emphasized, allowing individuals to withdraw at any time without repercussions. To maintain confidentiality, each participant was assigned a unique identification code, ensuring anonymity throughout the research process.

### 3.2 Data Collection and Analysis

A total of three focus group (FG) sessions were conducted between June and July 2024, each lasting approximately 60 min. The discussions explored key elements relevant to each group of participants. For dental students, the focus was on their motivations to participate in the DDC intervention, their views on its impact on their learning experience, their confidence and engagement skills in delivering the intervention, and their strategies to address oral health inequalities among vulnerable groups.

Supervisors discussed their perspectives on the project's effectiveness in enhancing student self‐efficacy, the support structures in place, and the long‐term influence of such interventions on students' professional development. Third‐sector managers provided insights on the intervention's relevance to their service users, the perceived impact on oral health awareness, and recommendations for improvement. The FG discussions were recorded via Microsoft Teams and transcribed by the PI.

The transcripts were then uploaded into NVivo software [23] for analysis using thematic analysis. The process followed Braun and Clarke's (2006) six stages: data familiarisation, initial coding, theme identification, theme review, theme definition, and report writing. Two researchers (MH and AR) independently coded the transcripts before meeting to discuss and resolve any discrepancies through consensus. Data was securely stored on the University's OneDrive with restricted access, and personal data was anonymized upon collection. All data will be retained for 5 years before being securely destroyed in compliance with data protection legislation.

## 4 Results

The analysis revealed seven major themes:

1. Enhancement of student competence and confidence.
2. Support structures and training programs.
3. Evolving Attitudes Towards Marginalised Groups.
4. Community Interaction and Preventive Dentistry.
5. Engagement and Motivation of Students.
6. Challenges and Strategies in Community Engagement.
7. Sustainability.

### 4.1 Enhancement of Student Competence and Confidence

**FIGURE 2:**
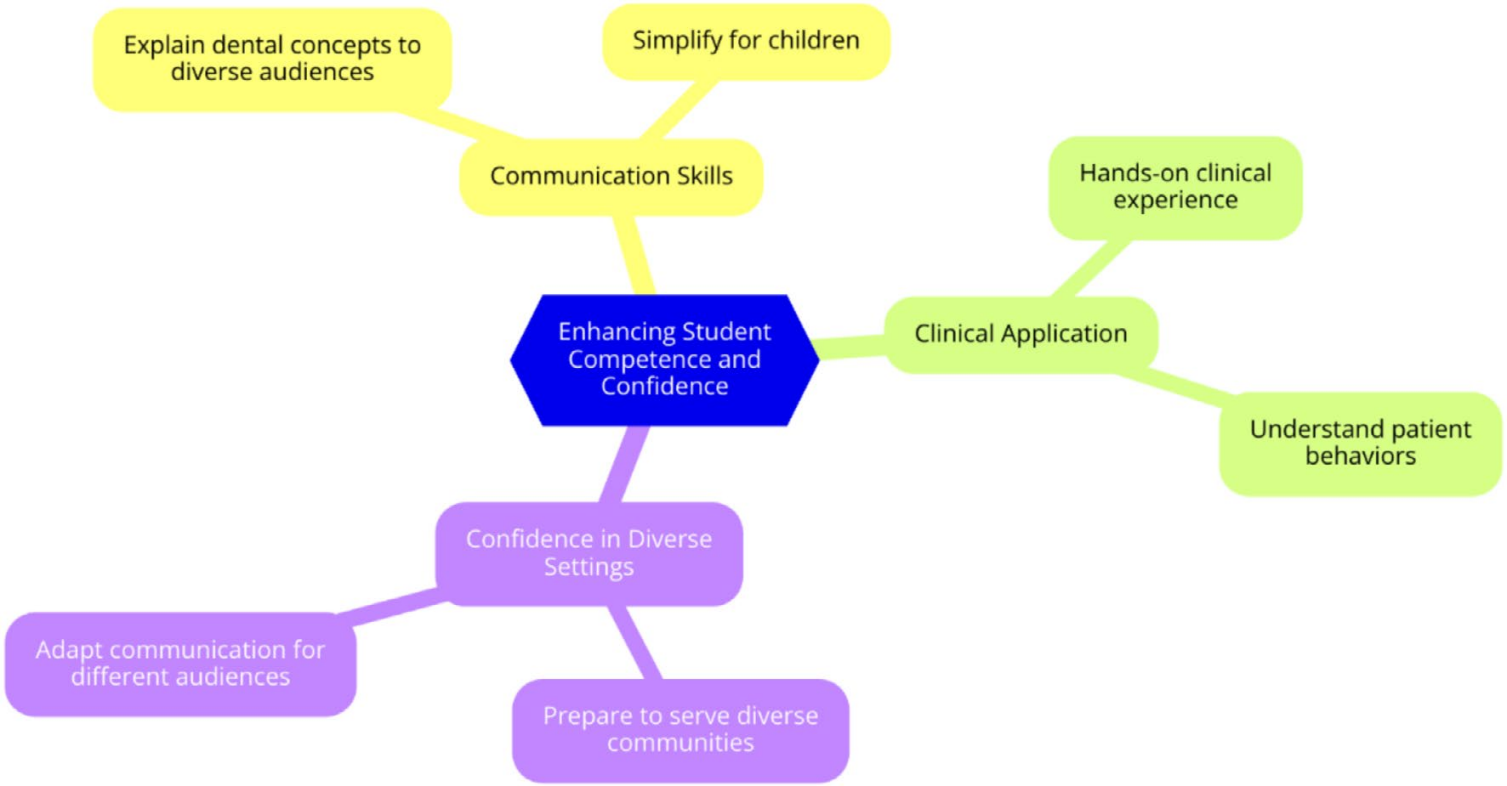
Results of theme 1, enhancement of student competence and confidence.

Students reported perceived significant improvements in professional competencies, particularly in communication skills, clinical application and confidence in diverse settings. One participant noted, “The school visits enhanced my ability to simplify complex dental concepts for children.” Another commented on the practical benefits: “When I go on to the pediatric clinic, I can understand kids' mannerisms better.” These experiences prepared students to serve diverse communities effectively, highlighting the project's value in bridging theoretical learning and real‐world application (Figure 2).

### 4.2 Support Structures and Training Programs

The project's success in fostering student self‐efficacy is attributed to comprehensive support structures and training, with three crucial elements emerging: mentorship, peer collaboration and effective training.

Mentorship played a key role. One supervisor noted, “For the school visits, students compile the information and put together the presentation, then staff like me go over what they've prepared and suggest improvements.” Another added, “The support structures ensure that students have the confidence of having staff involved in all of the projects.”

Peer collaboration was highly valued. A student commented, “I got a lot of help from my peers, which was useful in making these presentations.” Another emphasized, “Hearing positive feedback and what went well and what didn't go well at the primary schools from people who had done the visits before you, which is helpful.”

The effectiveness of training programs was crucial. A supervisor stated, “While it is student‐led, staff oversight ensures that all content in terms of slides, facts and regulatory advice is appropriate.” A student reflected, “We got to kind of watch how other people presented, and you kind of learned a few things from our peers.”

“The support structures ensure that students have the confidence of having staff involved in all of the projects.” Another supervisor added, “We also meet with the students to ensure they are calibrated and able to communicate appropriately, covering all relevant advice.”

**FIGURE 3:**
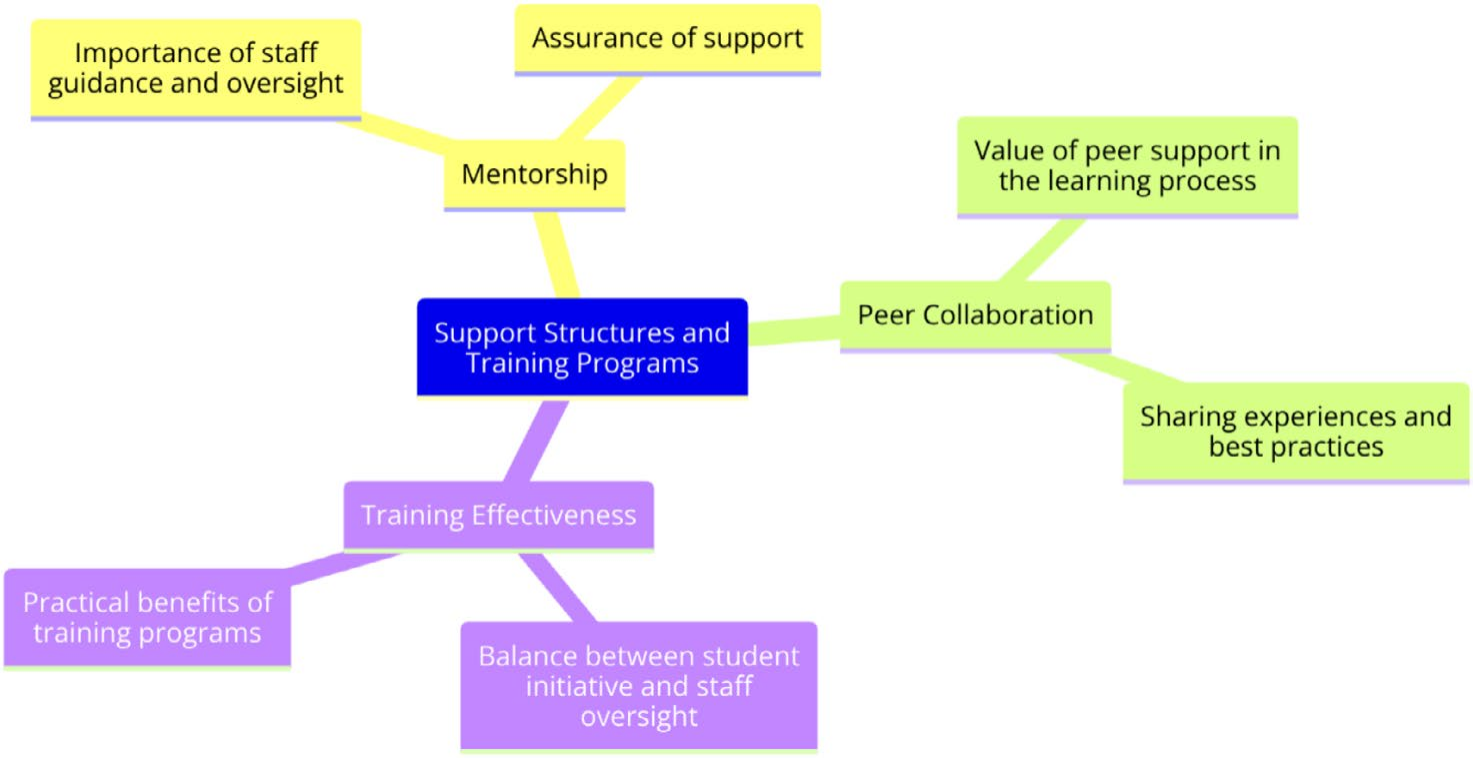
Result of theme 2, support structures and training programs.

These findings highlight the multifaceted support needed to build student self‐efficacy in community engagement projects (Figure 3).

### 4.3 Evolving Attitudes Towards Marginalised Groups

DDC (Dundee Dental Connect) has significantly influenced students' perspectives and attitudes towards marginalised communities, fostering empathy, understanding systemic barriers and appreciation for preventive care—crucial for addressing oral health disparities.

Students reported profound experiences enhancing their empathy. One shared, “I remember going to the food bank… you got a real chance to speak to people, kind of find out people's struggles.” Another recalled a child who had never visited a dentist: “One of the kids… said he's never been to the dentist, or his mom and dad never took him… this was the first time he was experiencing it.”

**FIGURE 4:**
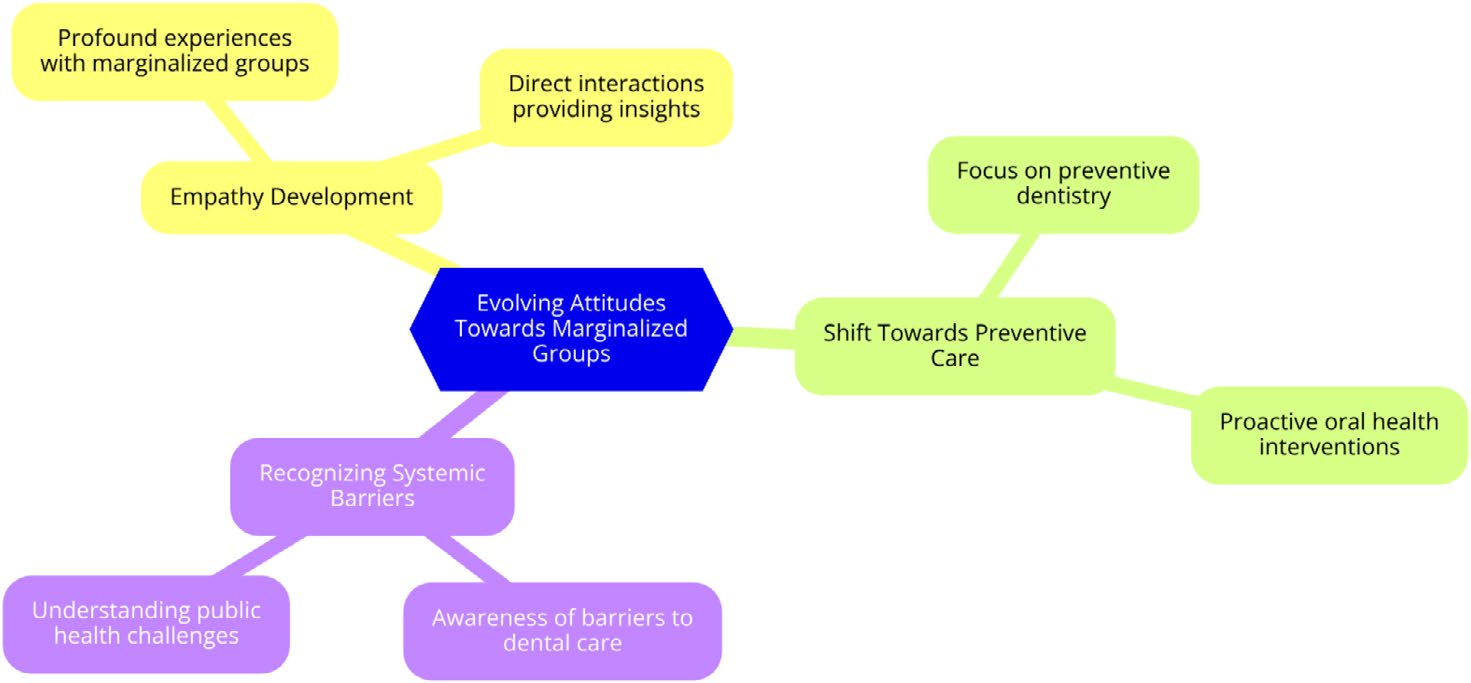
Result of theme 3, evolving attitudes towards marginalised groups.

Students gained awareness of systemic barriers to dental care. One noted, “Some parents might not know how important it is to take the kids to the dentist. So, I think that just sort of highlighted to me how important it is to keep doing these visits.” Another observed, “It's highlighted the importance of the patient's attitude towards dental care and oral health… to prevent dental anxiety across families.” (Figure 4).

### 4.4 Community Interaction and Preventive Dentistry

The DDC project has highlighted the crucial link between community engagement and preventive dentistry, emphasizing trust‐building and a community‐centred approach in dental education.

Building trust was vital. A third‐sector manager noted, “Engaging with our clients is brilliant.” Another participant emphasized, “It's important to build trust and engage interest from the community.”

Preventive dentistry was a key focus. One student stated, “If we can prevent dental caries and disease, then it's less likely they'll be in pain and struggling to go to the dentist.” Another reflected, “It's been amazing to have a positive impact on the lives of some of the children in Dundee, and it's heightened my appreciation of the importance of preventive dentistry.”

A supervisor explained, “For students, focusing wholly on prevention in a supportive environment helps them develop communication skills essential for conveying preventive messages.” Community impact was evident, with a third‐sector manager observing, “The clients who did engage and I think the students who were here were very pleased with the amount of engagement they received from the clients at the Food Bank.”

One student summarised, “I think projects like these are the future of dentistry. As prevention is the future of dentistry.” These findings suggest DDC is nurturing dental professionals who understand the relationship between trust, prevention and long‐term oral health, shaping future dental practice.

**FIGURE 5:**
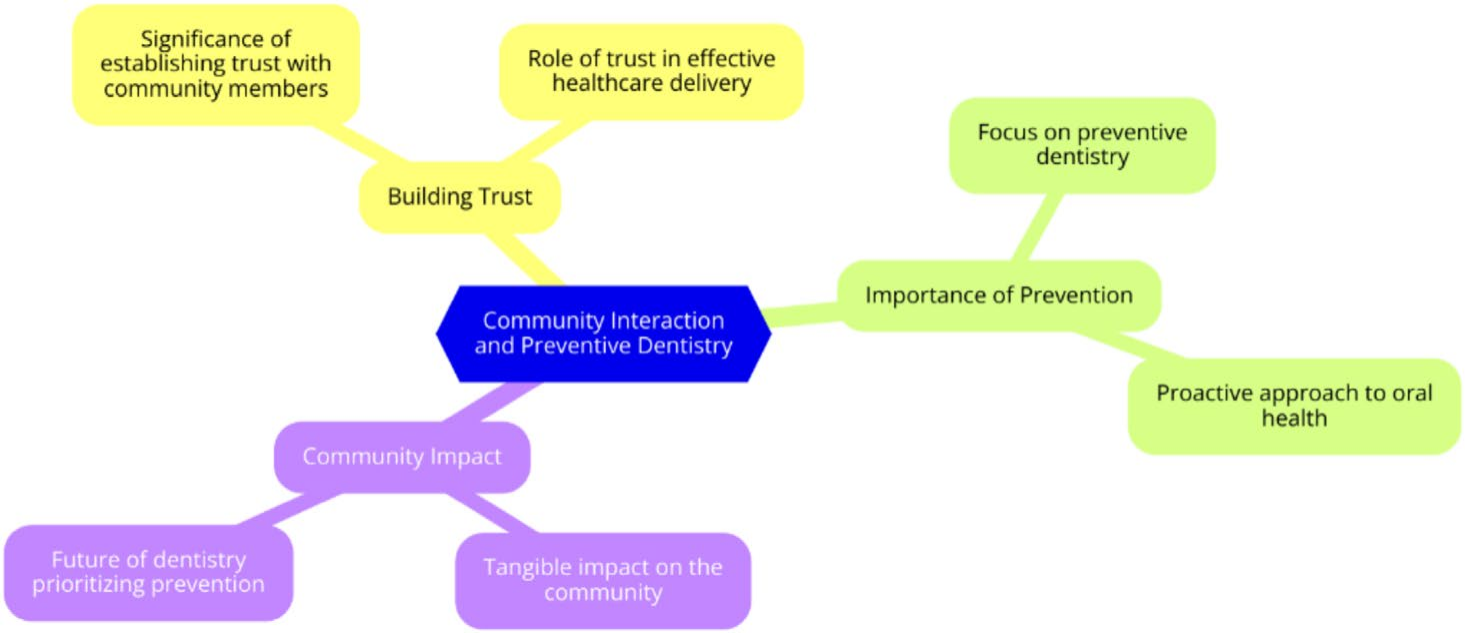
Result of theme 4, community interaction and preventive dentistry.

Feedback from the ESOL family provided further insights into the impact and future directions for the project. A representative from Dundee City Council's CLD Service commented, “Our partnership with DDC is a very important and valuable one as it helps our learners gain knowledge and skills in the vital area of oral hygiene and learn topic‐specific words and expressions in English at the same time.” They highlighted the proactive nature of the students, noting, “The dental students have been very proactive, enthusiastic, flexible, well‐prepared, and open to feedback.” (Figure 5).

### 4.5 Engagement and Motivation of Students

The DDC project demonstrated high student engagement and motivation highlighting the potential of community‐based learning to enhance satisfaction and commitment to dental education.

Personal motivation played a key role. One student stated, “High school volunteering inspired a lasting commitment to community engagement at university.” Another shared, “Dental Connect has been a really positive part of my university life,” reflecting the project's valued role in their education.

Student reflections showed evolving engagement. One noted, “The initial draw for me was Toothy Tigers [19] in terms of the primary school visits because that sparked the beginning of Dental Connect. We've had different projects alongside it.” Her continued interest in “volunteering alongside children and linking education and dentistry” illustrates alignment with student interests.

**FIGURE 6:**
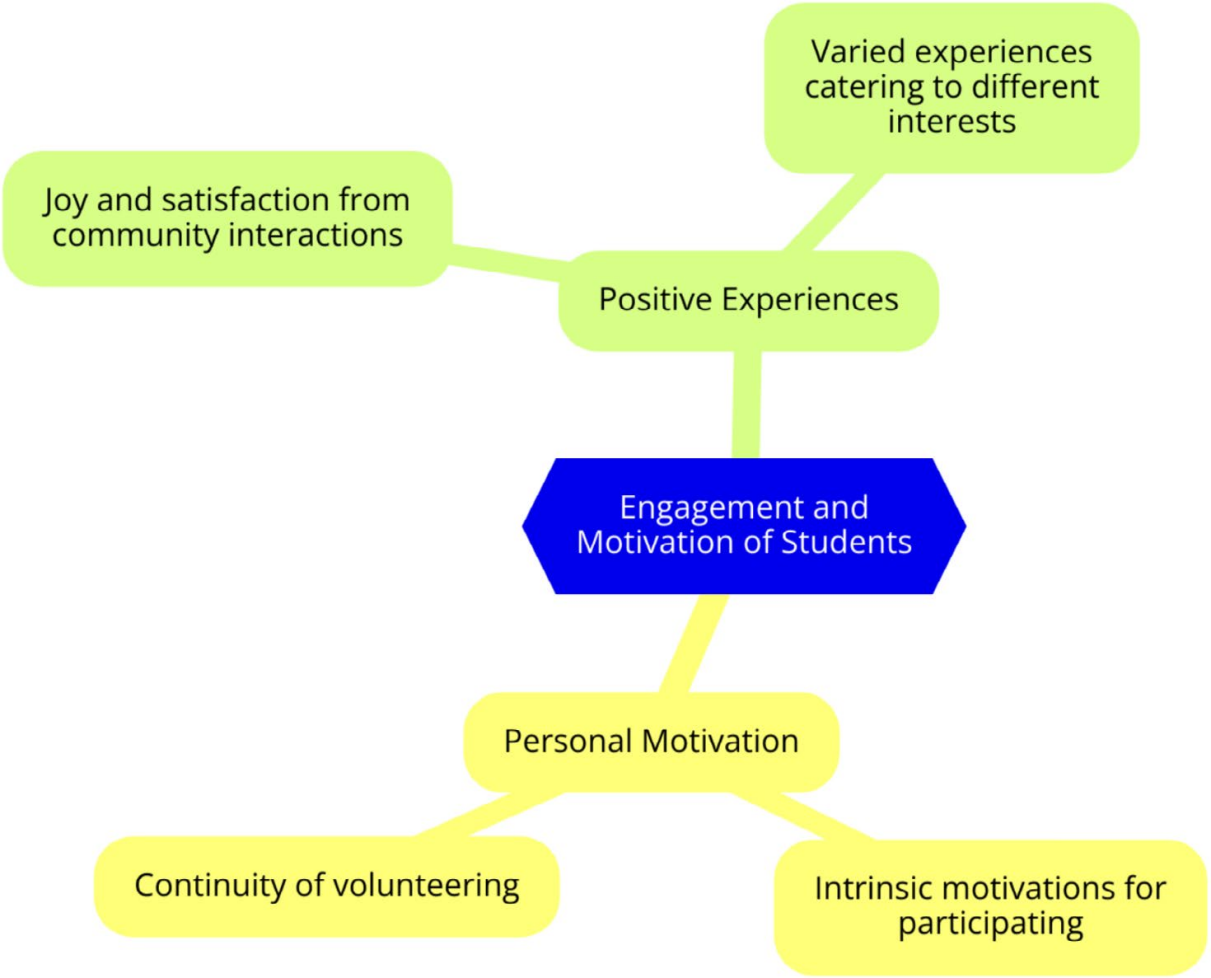
Result of theme 5, engagement and motivation of students.

Positive experiences reinforced engagement. One student remarked, “It was so nice to see all the kids. They were super engaging, enjoying participating, and asking questions, which was lovely to see.” Another's enjoyment in fundraising activities highlighted the project's diverse opportunities (Figure 6).

### 4.6 Challenges and Strategies in Community Engagement

The Dundee Dental Connect (DDC) project demonstrated development of insight into the significant challenges in community engagement strategies, highlighting the complexity of community‐based oral health education and the importance of adaptability and problem‐solving for future professionals.

Adapting to diverse needs was essential. A supervisor noted, “One challenge is dealing with a wide range of ages and conditions. For instance, older patients may be edentulous or have poor dentures, requiring students to think on their feet and adjust their advice accordingly.”

Barriers in engagement were prevalent. One student highlighted the language barrier with Ukrainian refugees: “An obvious challenge is the language barrier, and many couldn't understand us when chatting about signing up for treatment at the dental hospital.” Visual aids and translated materials helped overcome this. Another shared their approach to managing large groups of children: “Some got upset when others played with models before them, but overcoming that was just a case of saying, ‘Oh yeah, we'll do it for two minutes and then you guys will get a shot.’ It's about staying calm and having a smile on your face.”

**FIGURE 7:**
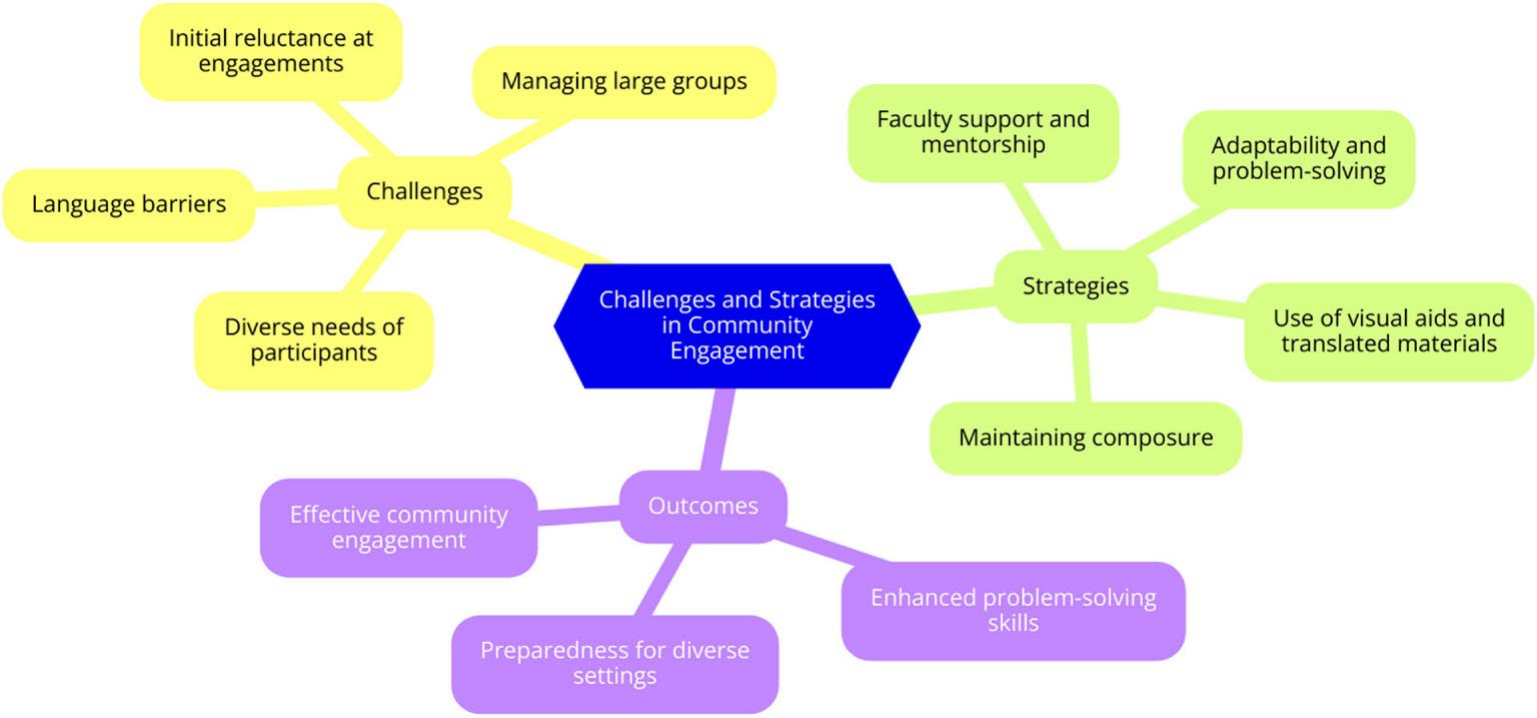
Result of theme 6, challenges and strategies in community engagement.

Effective strategies were key. One student observed initial reluctance at food bank visits: “Initially, at some food bank visits, people didn't really want to come over or chat.” Another emphasized faculty support's role, noting the value of mentorship in navigating challenges (Figure 7).

### 4.7 Project Sustainability and Future Recommendations

The final theme focuses on the sustainability of the Dundee Dental Connect (DDC) project and recommendations for its future development, emphasizing resource management, scheduling, outreach expansion and enhanced training. Students had insight into project management.

Effective resource management emerged as a key to sustainability. One student highlighted ongoing efforts: “I'm always contacting companies and organizations and trying to get resources donated.”

Participants identified opportunities for expanding outreach. One student suggested, “There's also a wider scope that we can always go out to, but it includes nurseries or care homes.” Another student observed current limitations regarding resources: “I think that is kind of one of the main limitations. Like I think if we had a lot more, we could be going out to do visits even more often than we already are.”

Another student expressed an aspirational vision: “We hope that by implementing such an initiative in Dundee, we inspire dental students across the UK to do something similar at their dental school. Because I think when you come to dental school, we realize that we are coming away from reactionary dentistry where you just treat the disease; it is all about prevention and stopping the disease from occurring. So, I think projects like these are the future of dentistry. As prevention is the future of dentistry.”

Areas for improvement were also noted, such as involving learners more actively and ensuring content understanding. The representative suggested, “I think the students would benefit from more guidance on how to involve the learners in the sessions and ensure understanding of the content.” They recommended collaboration between ESOL tutors and dental students to design session plans that integrate CLIL (Content and Language Integrated Learning) principles, making sessions more interactive and effective.

**FIGURE 8:**
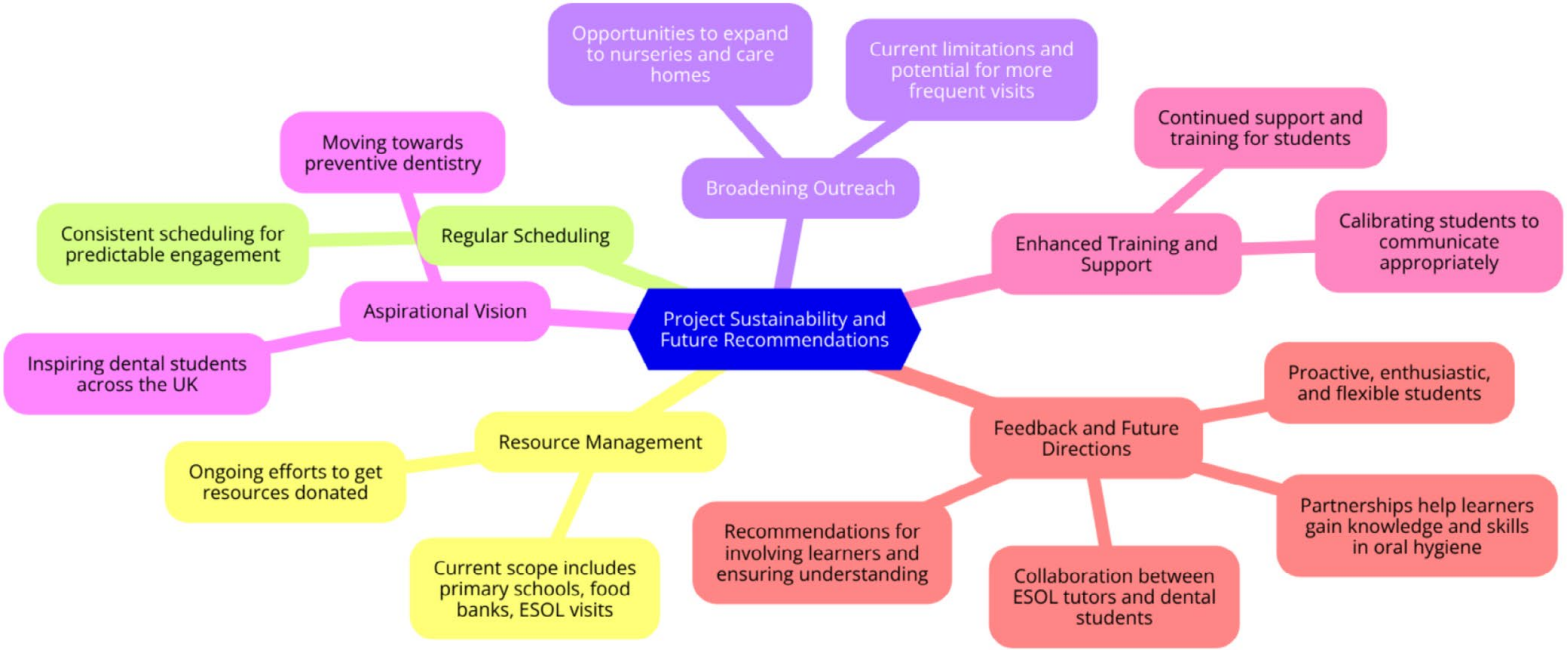
Result of theme 7, project sustainability and future recommendations.

These recommendations provide a roadmap for the project's future development, emphasizing sustainable resource management, consistent community engagement, expanded outreach and ongoing training (Figure 8).

## 5 Discussion

The DDC project has demonstrated a significant impact on dental education and community oral health through its innovative co‐creation approach. The study revealed seven key themes that highlight the project's multifaceted benefits. Notably, student participants' reported increased confidence in their ability to communicate effectively with diverse patient groups, as they had to simplify complex dental concepts for individuals with varying levels of health literacy. This real‐world engagement improved their adaptability and problem‐solving abilities, skills that are essential for future dental practice. The project's structured mentorship and peer collaboration further enhanced student self‐efficacy, reinforcing their ability to educate and interact with vulnerable populations.

Beyond student development, the DDC project also positively impacted community oral health by increasing awareness and access to preventive care. Community members engaged in the project, such as those in food banks and ESOL (English for Speakers of Other Languages) programs, received tailored oral health advice and resources that directly addressed their needs. Feedback from third‐sector managers indicated that participants found the sessions highly beneficial, particularly in helping them understand the importance of preventive dental care.

These findings align with previous research on co‐creation in dental education. Campbell et al. found that involving dental students in co‐creation improved their confidence and professional skills [28], while Könings et al. highlighted the importance of active learner involvement in medical education [29]. Billett and Martin explored the impact of student‐staff partnerships in curriculum design, highlighting how co‐creation fosters deeper engagement, enhances learning experiences and empowers students in the educational process [30]. Successful applications of co‐creation in healthcare education include developing VR resources [31], enhancing digital resources for nursing students [32] and implementing creative co‐design for knowledge mobilisation [33].

Co‐design and co‐creation methodologies in higher education are promising strategies that can revolutionise both education and community health interventions [34, 35]. Recent systematic reviews have identified multiple benefits of student co‐creation in higher education, including enhanced engagement, improved learning outcomes and development of professional skills [36]. By involving students as active partners in their education and in community health initiatives, institutions can create more relevant, meaningful and impactful learning experiences [37, 38].

This approach bridges the gap between theoretical knowledge and practical application [39]. When students work closely with their supervisors, it promotes mutual respect, understanding and a shared commitment to improving health outcomes [40, 41]. This collaborative approach can transform the learning process [42], encouraging a more personalised and engaging learning journey [43, 44]. Co‐design practices can lead to innovative educational strategies [45, 46], enriching the academic environment and making it more relevant to modern healthcare delivery [47, 48].

A notable outcome was the transformation in students' attitudes towards marginalised groups. Through participation in the DDC project, students reported increased empathy, a greater appreciation of the challenges faced by these communities, and a more proactive approach to addressing oral health disparities. Many students highlighted their previous limited awareness of systemic barriers affecting access to dental care. The project's emphasis on community interaction and preventive dentistry reinforced a shift towards a more holistic, community‐centered approach in dental education.

The results indicate a significant improvement in students' professional competencies and self‐confidence, aligning with Bandura's self‐efficacy theory [49]. These findings are consistent with Strauss et al. [50], which highlighted that community‐based dental education programs significantly improve students' clinical skills, cultural competence and professional development. Similarly, Orsini et al. [51] emphasised the role of intrinsic motivation in fostering better learning outcomes and professional growth in health professions education.

The project's impact aligns with transformative learning in health professions education, as described by Mezirow and Taylor [52] and reflects the World Health Organization's advocacy for integrating oral health into primary health care [53]. Formicola and Bailit [54] found that community‐based dental education programs enhance understanding of public health issues and commitment to serving underserved populations. The high levels of student engagement and motivation observed in the DDC project are crucial for effective learning and skill development, as noted by Perez et al. [55].

## 6 Limitations

While this study provides valuable insights, it is important to acknowledge its limitations. The qualitative approach and small sample size limit generalizability to broader dental education settings and student populations. In addition, despite the study captures immediate impacts on students' self‐efficacy and attitudes it does not address long‐term effects on professional practices and lacks perspectives from service users who received the oral health interventions.

Future research could benefit from a mixed‐methods approach, incorporating quantitative measures alongside qualitative insights. Longitudinal studies tracking students' career choices and practice patterns post‐graduation would provide valuable information on the long‐term impact of such interventions on addressing oral health disparities.

## 7 Conclusion

The DDC project demonstrates the significant benefits of integrating a co‐creation approach into dental education to address oral health disparities in marginalised communities. Through this innovative model, dental students not only enhanced their professional competencies and self‐confidence but also developed a deeper understanding and empathy towards underserved populations. The project highlighted the importance of support structures, such as mentorship and peer collaboration, in fostering student self‐efficacy and effective community engagement.

The study revealed that community‐based learning, focused on preventive dentistry and building trust within communities, can shift dental education towards more holistic and sustainable practices. High levels of student engagement and motivation, coupled with the ability to navigate real‐world challenges, underscore the potential of such initiatives to prepare future dental professionals for diverse and complex healthcare environments.

The study emphasises the need for ongoing research to explore long‐term impacts and educational strategies that incorporate the voices of marginalised communities by continuing to innovate and adapt interventions using co‐creation and co‐design approaches.

Recommendations for project sustainability, including effective resource management and expanded outreach, highlight the potential scalability of the DDC project as a model for other dental schools. Dental education enhanced by oral health interventions such as the DDC can play a pivotal role in promoting health equity and fostering a new generation of socially responsible healthcare professionals. The findings contribute to a growing body of evidence supporting the integration of community‐based and co‐creation approaches in dental education, aiming to reduce oral health inequalities and improve overall community well‐being.

## Ethics Statement

The study received ethical approval from the University of Dundee School Research Ethics Committee (UOD‐SREC‐SDEN‐2024‐008).

## Conflicts of Interest

One of the authors is a founding member of Dundee Dental Connect. This could potentially be seen as a conflicts of interest. However, the author was not involved in the recruitment of participants, in the data collection or the analysis/interpretation. Therefore, their involvement does not bias the results.

## Data Availability

The data that support the findings of this study are available on request from the corresponding author. The data are not publicly available due to privacy or ethical restrictions.
